# Combining deep learning with statistical shape modelling enables automated lower limb measurements with observer‐level reliability using weight‐bearing computed tomography

**DOI:** 10.1002/jeo2.70669

**Published:** 2026-02-26

**Authors:** Ide Van den Borre, Emmanuel Audenaert, Hannes Vermue, Roel Huysentruyt, Leonie Van Vynckt, Robin Vanhauwe, Victor Pas, Aleksandra Pizurica, Aline Van Oevelen

**Affiliations:** ^1^ Department of Telecommunications and Information Processing Ghent University Ghent Belgium; ^2^ Department of Human Structure and Repair Ghent University Ghent Belgium; ^3^ Department of Orthopaedic Surgery and Traumatology Ghent University Hospital Ghent Belgium; ^4^ Department of Electromechanics University of Antwerp Antwerp Belgium

**Keywords:** deep learning, lower limb alignment, medical image analysis, statistical shape modelling, Weight‐bearing CT

## Abstract

**Purpose:**

Accurate anatomical landmarking is crucial for assessing lower limb alignment, diagnosing deformities, planning surgeries and monitoring treatment outcomes. Traditional methods rely on manual measurements from 2D standing radiographs, which fail to capture 3D bone morphology and are influenced by patient positioning. Weight‐bearing computed tomography (WBCT) enables 3D evaluations under physiological loading conditions, but manual landmark identification on WBCT is time‐consuming and subject to observer variability. This study aims to leverage deep learning (DL) and statistical shape modelling (SSM) for automated assessment of lower limb alignment and morphology.

**Methods:**

A hybrid DL‐SSM model automatically calculated 28 lower limb alignment and morphology measurements using 30 full‐leg WBCT scans. The DL model was trained in a five‐fold cross‐validation setting. It automatically segmented the femur, patella, tibia, talus, calcaneus and second metatarsal. A cascaded SSM‐fitting methodology automatically identified the necessary 3D landmarks to derive the 28 measurements. The automated measurements were statistically compared to manual measurements performed by three experienced raters on both WBCT scans and 3D bone models.

**Results:**

The DL segmentation model achieved high accuracy, with a mean dice similarity coefficient exceeding 0.96. The proposed method corresponded well to manual assessments, with the magnitude of detected differences generally matching the interobserver reliability of the manual method. The mean absolute error for the angular measurements ranged from 0.35° ± 0.39° to 5.53° ± 4.68°.

**Conclusion:**

The hybrid DL‐SSM methodology for automated assessment of lower limb alignment demonstrated comparable reliability to manual methods. This method provides an observer‐independent method for 3D lower limb alignment and morphology assessment under weight‐bearing conditions.

**Level of Evidence:**

Level III.

**Abbreviations**
CT: computed tomography
DL: deep learning
DSC: dice similarity coefficient
HD: hausdorff distance
ICC: intraclass correlation coefficient
ICP: iterative closest point
MAE: mean absolute error
PCA: principal component analysis
SSM: statistical shape modelling
WBCT: weight‐bearing computed tomography

## INTRODUCTION

Anatomical landmarking for precise angular and linear measurements is a critical aspect of orthopaedics, used to assess alignment, detect deformities, plan surgical interventions and monitor treatment progress. This process is particularly significant in preoperative planning of knee surgeries [39]. These surgeries include osteotomies to correct malalignment or posttraumatic deformities [35]. Moreover, in joint replacement surgery, accurate identification of anatomical landmarks serves as a reliable reference point for surgeons to precisely position implants.

Traditionally, knee alignment evaluation has relied primarily on manual measurements performed on 2D standing radiographs. While these methods provide valuable information, they possess inherent limitations. These radiographic images cannot capture the full 3D dynamic nature of knee alignment and morphology during weight‐bearing. Furthermore, variations in patient positioning during radiographic imaging can lead to alterations in the apparent osseous alignment, potentially affecting measurement accuracy [1, 28].

In recent years, the advent of cone‐beam weight‐bearing computed tomography (WBCT) imaging has enabled the evaluation of 3D lower limb alignment and morphology under physiological loading conditions [11, 14, 25]. This allows for a more comprehensive understanding of the complex interactions between the femur, tibia and patella. However, the process of manual landmark selection on 3D imaging data is time‐consuming and prone to observer variability [31].

Consequently, there is growing interest in automated anatomical landmark identification directly from medical imaging data [23, 32, 34]. A popular and established approach involves anatomical landmark transfer from expert‐annotated bone models. This can be achieved either by incorporating prior anatomical knowledge through statistical shape models (SSMs) or by applying nonrigid deformation of an annotated template [12, 30]. Deformation‐based landmark transfer has been extensively used across various application domains, partly due to the early open‐source availability of registration algorithms such as the nonrigid Iterative Closest Point (ICP) framework introduced by Audenaert et al. in 2013 [3]. For example, this framework was employed in a recent study by Kuiper et al., who reported promising results using nonrigid ICP‐based landmark transfer [23]. However, the original authors of the aforementioned nonrigid ICP algorithm have demonstrated and advocated improved accuracy when such deformation techniques are combined with prior anatomical knowledge derived from statistical shape modelling (SSM) [3, 9, 13]. When combined, they hold significant potential for delivering standardised, reliable and accurate measurements using computer algorithms.

These template deformation approaches require accurate patient‐specific 3D bone surfaces. The rise of deep learning (DL) has revolutionised this step, enabling fast and precise 3D bone segmentation from medical imaging data and thereby paving the way for clinical applications [6, 17, 22]. This progress has enabled the development of fully automated pipelines that integrate image segmentation with downstream landmark transfer and measurement extraction. Kuiper et al. introduced one of the first comprehensive and fully automated workflows for quantifying 25 lower limb alignment parameters from computed tomography (CT) data by combining a DL segmentation model with a nonrigid landmark transfer methodology [23]. Their method was developed and validated using conventional CT acquisitions, which implies that the reported alignment measurements were derived under supine conditions. Consequently, these measurements may differ from those obtained under physiological loading, as previously reported in the literature [14, 24, 26]. In addition, patellofemoral measurements were not included in their study, despite their importance for assessing patellofemoral instability and patellofemoral kinematics.

This study proposes a hybrid DL‐SSM methodology for fully automated quantification of 28 distinct knee and ankle measurements, including 3 patellar, 20 knee and five ankle parameters, directly from WBCT images. This hybrid approach combines a state‐of‐the‐art DL segmentation model for automated bone segmentation, with SSMs to incorporate prior anatomical shape constraints for automated landmark transfer. The model was validated by comparing the automated measurements against measurements derived from manually identified landmarks by three expert raters.

The objectives of this study are threefold:

1. To evaluate the accuracy of a DL‐based framework for automated bone reconstruction from WBCT images.
2. To assess the reliability and accuracy of automated measurements derived from the proposed cascaded SSM‐based landmark transfer methodology.
3. To compare manual measurements performed on 3D WBCT images with those performed on 3D bone models.

## MATERIALS AND METHODS

### Study population and design

In this retrospective cohort study, 30 patients who underwent a full‐leg WBCT were included. The dataset consisted of 16 females and 14 males with an age range of 18 to 72 years (mean age: 41.3 ± 15.1 years). Imaging data were obtained from the HiRise WBCT imaging device (CurveBeam). Inclusion required a WBCT scan of the lower limb acquired between 2022 and 2024. The exclusion criteria included a history of previous surgery, the presence of orthopaedic hardware, osteoarthritis classified as Kellgren–Lawrence grade 2, 3 or 4, and age below 18 or above 75 years.

The patients were positioned with both feet parallel at shoulder width and were instructed to maintain full extension at the knee. The HiRise was used at the following settings: tube voltage: 130 kV; tube current: 6.5 mAs; pixel size: 0.5 mm; slice thickness: 0.5 mm. The estimated radiation dose per scan was approximately 1.5 mSv and confined to the lower extremities, thereby avoiding exposure of radiosensitive organs such as the breasts, thyroid and abdomen.

The femur, patella, tibia and fibula, talus and calcaneus of the 30 WBCT images were manually segmented by a fellowship‐trained orthopaedic surgeon using Materialise Mimics 21.0 (Materialise NV). These served as the ground truth for training the DL segmentation model. The manual segmentations were visually inspected and iteratively cross‐checked by two independent orthopaedic specialists before model training.

### Automated measurements

**Figure 1:**
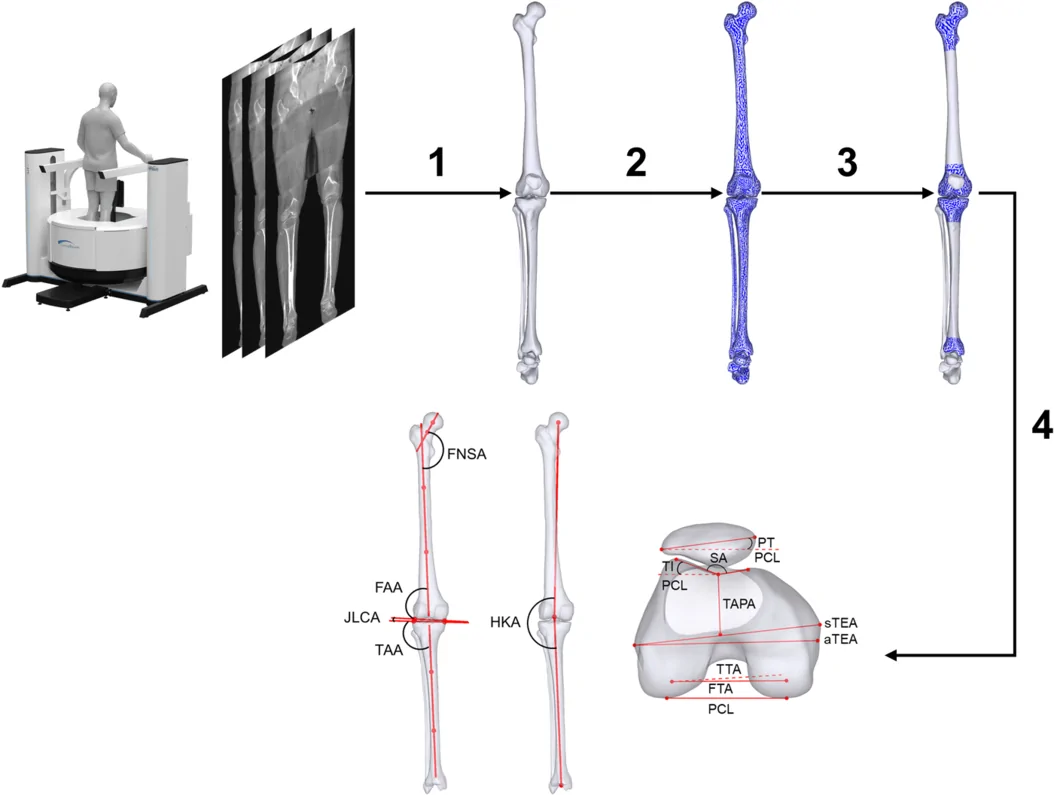
Proposed DL‐SSM hybrid methodology to automatically assess lower limb alignment directly from WBCT images. The process involves: (1) automated segmentation of the femur, patella, tibia, fibula, talus and calcaneus bones using the nnU‐Net model; (2) global SSM fitting on the DL‐based reconstruction; (3) local proximal/distal femur and tibia SSM fitting; and (4) final landmark transfer and calculation of the orthopaedic measurements. aTEA, anatomical transepicondylar axis; DL, deep learning; FAA, femoral anatomical angle; FNSA, femoral neck‐shaft angle; FTA, femoral transverse axis; HKA, hip‐knee‐ankle angle; JLCA, joint line convergence angle; PCL, posterior condylar line; PT, patellar tilt; SA, sulcus angle; SSM, statistical shape modelling; TAA, tibial anatomical angle; TAPA, trochlear antero‐posterior axis; TT, talar tilt; TTA, tibial transverse axis; WBCT, weight‐bearing computed tomography.

**Figure 2:**
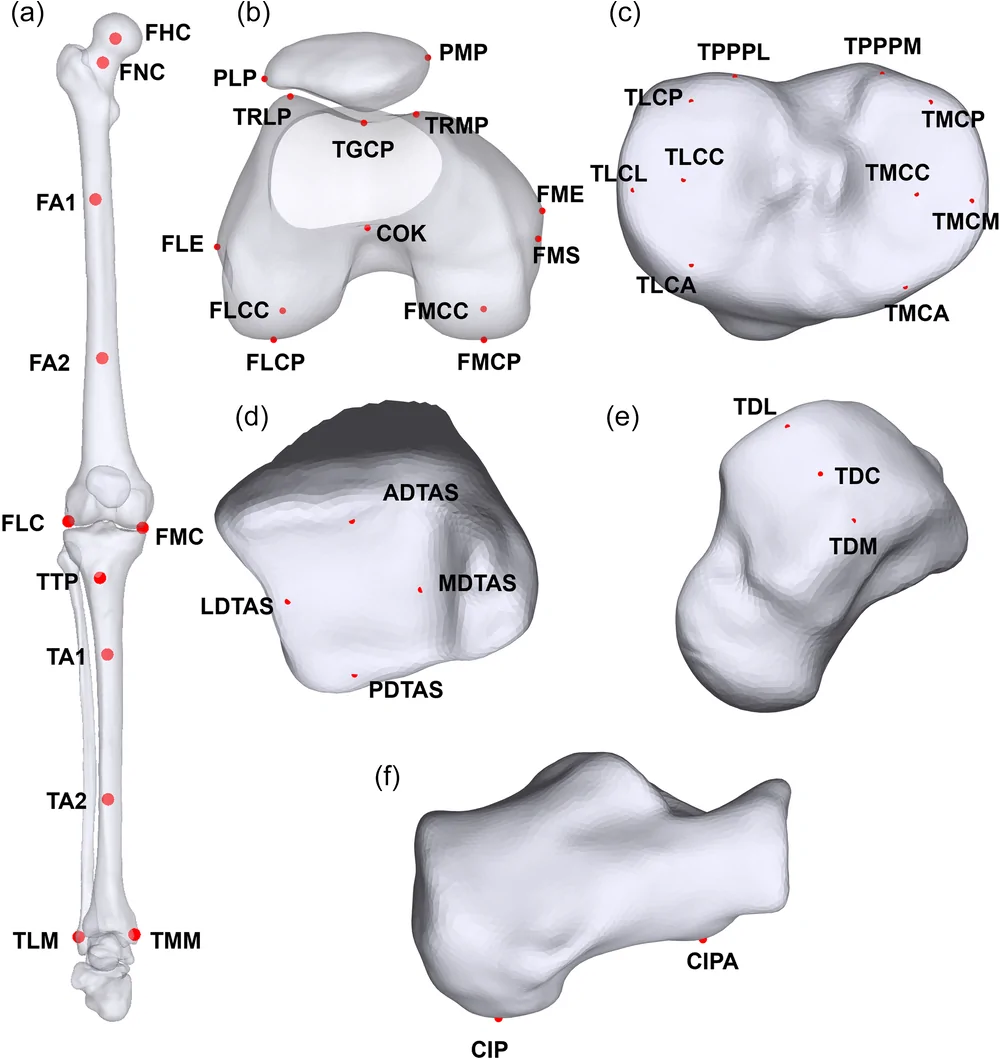
Different landmarks required for the automated measurements on the (a) complete femur, patella, tibia, fibula, talus and calcaneus; (b) distal femur and patella; (c) proximal tibia; (d) distal tibia; (e) talus; and (f) calcaneus. ADTAS, anterior distal tibial articular surface; CIP, calcaneal inferior point; CIPA, calcaneal inferior point anterior; COK, centre of knee; FA1, femoral axis 1; FA2, femoral axis 2; FHC, femoral head centre; FLC, femoral lateral condyle; FLCC, femoral lateral condyle centre; FLCP, femoral lateral condyle posterior; FLE, femoral lateral epicondyle; FMC, femoral medial condyle; FMCC, femoral medial condyle centre; FMCP, femoral medial condyle posterior; FME, femoral medial epicondyle; FMS, femoral medial sulcus; FNC, femoral neck centre; LDTAS, lateral distal tibial articular surface; MDTAS, medial distal tibial articular surface; PDTAS, posterior distal tibial articular surface; PLP, patella lateral pole; PMP, patella medial pole; TA1, tibial axis 1; TA2, tibial axis 2; TDC, talar dome centre; TDL, talar dome lateral; TDM, talar dome medial; TGCP, trochlear groove central point; TLCA, tibial lateral condyle anterior; TLCC, tibial lateral condyle centre; TLCL, tibial lateral condyle lateral; TLCP, tibial lateral condyle posterior; TLM, tibial lateral malleolus; TMCA, tibial medial condyle anterior; TMCC, tibial medial condyle centre; TMCM, tibial medial condyle medial; TMCP, tibial medial condyle posterior; TMM, tibial medial malleolus; TPPPL, tibial posterior proximal plateau lateral; TPPPM, tibial posterior proximal plateau medial; TRLP, trochlear ridge lateral point; TRMP, trochlear ridge medial point; TTP, tibial tuberosity point.

The entire workflow for obtaining the automated orthopaedic measurements is visualised in Figure 1. First, the lower limb bones were automatically segmented from the WBCT images. Second, the necessary anatomical landmarks for computing the measurements were transferred using SSM fitting. The final landmark transfer was obtained after nonrigid ICP deformation of the SSM‐fitted shapes [3]. Table 1 provides an overview of the anatomical landmarks used in this study for the calculation of the different measurements, which are also visualised in Figure 2.

**Table 1:** The 45 landmarks required for the automated measurements.

Landmark	Acronym	Definition
Femoral head centre^a^	FHC	Centre of sphere fitted to the femoral head vertices
Femoral neck centre^a^	FNC	Midpoint of the vertices of the femoral neck.
Femoral axis 1–2^a^	FA1–FA2	Midpoints of surface vertices in the proximal (FA1) and distal (FA2) femoral shaft regions.
Femoral lateral condyle	FLC	Most distal point of the femoral lateral condyle
Femoral medial condyle	FMC	Most distal point of the femoral medial condyle
Femoral medial condyle posterior	FMCP	Most posterior point of the femoral medial condyle
Femoral lateral condyle posterior	FLCP	Most posterior point of the femoral lateral condyle
Femoral medial condyle centre	FMCC	Centroid of surface vertices comprising the medial condylar region
Femoral lateral condyle centre	FLCC	Centroid of surface vertices comprising the lateral condylar region
Centre of knee	COK	Most posterior point of the trochlear groove
Femoral lateral epicondyle	FLE	Most lateral point of the lateral epicondyle
Femoral medial epicondyle	FME	Most medial point of the medial epicondyle
Femoral medial sulcus	FMS	Deepest point of the medial epicondylar sulcus
Trochlear ridge lateral point	TRLP	Most prominent point on the lateral trochlear ridge of the distal femur
Trochlear ridge medial point	TRMP	Most prominent point on the medial trochlear ridge of the distal femur
Trochlear groove central point	TGCP	Deepest point of the trochlear groove
Patella medial pole	PMP	Most medial point on the patellar surface or contour
Patella lateral pole	PLP	Most lateral point on the patellar surface or contour
Tibial lateral condyle lateral	TLCL	Most lateral point of the lateral tibial condyle
Tibial lateral condyle anterior	TLCA	Most anterior point of the lateral tibial condyle
Tibial lateral condyle posterior	TLCP	Most posterior point of the lateral tibial condyle
Tibial lateral condyle centre	TLCC	Centre of the lateral tibial condyle surface
Tibial medial condyle medial	TMCM	Most medial point of the medial tibial condyle
Tibial medial condyle anterior	TMCA	Most anterior point of the medial tibial condyle
Tibial medial condyle posterior	TMCP	Most posterior point of the medial tibial condyle
Tibial medial condyle centre	TMCC	Centre of the medial tibial condyle surface
Tibial posterior proximal plateau lateral	TPPPL	Most protruding point of the lateral tibial plateau
Tibial posterior proximal plateau medial	TPPPM	Most protruding point of the medial tibial plateau
Tibial tuberosity point	TTP	Most anterior and superior point on the tibial tuberosity
Medial distal tibial articular surface	MDTAS	Midpoint of the medial edge of the distal tibial articular surface
Lateral distal tibial articular surface	LDTAS	Midpoint of the lateral edge of the distal tibial articular surface
Anterior distal tibial articular surface	ADTAS	Midpoint of the anterior edge of the distal tibial articular surface
Posterior distal tibial articular surface	PDTAS	Midpoint of the posterior edge of the distal tibial articular surface
Tibial centre proximal^a^	TCP	Midpoint of TLCC and TMCC
Tibial axis 1–2^a^	TA1–TA2	Midpoints of surface vertices in the proximal (TA1) and distal (TA2) tibial shaft regions
Tibial lateral malleolus	TLM	Most lateral point of the lateral malleolus on the distal fibula
Tibial medial malleolus	TMM	Most medial point of the medial malleolus on the distal tibia
Tibial centre distal^a^	TCD	Midpoint between TMM and TLM
Talar dome lateral	TDL	Most lateral point on the superior articular surface of the talar dome
Talar dome medial	TDM	Most medial point on the superior articular surface of the talar dome
Talar dome centre^a^	TDC	Centre of the talar dome articular surface, defined as the midpoint between TDL and TDM
Calcaneal inferior point	CIP	Most inferior point on the plantar surface of the calcaneus
Calcaneal inferior point anterior	CIPA	Most anterior point along the inferior border of the calcaneus

^a^Means that these 3D landmarks were derived indirectly.

#### Automated bone segmentation

As the first step in the automated measurement pipeline, the femur, patella, tibia, fibula, talus, calcaneus and second metatarsal were automatically segmented from the WBCT images. A 3D nnU‐Net model was trained in a 5‐fold cross‐validation setting on 30 subjects for segmentation of the femur, patella, tibia, fibula, talus and calcaneus [17]. The metatarsal bone was automatically segmented from the WBCT images using a previously developed and validated DL–based foot bone segmentation model [6]. In each fold, the training set consisted of 24 cases, while 6 cases were reserved for validation. The nnU‐Net v2 framework was selected due to its state‐of‐the‐art performance in medical image segmentation and its proven generalisability across a wide range of biomedical imaging tasks. Unlike conventional DL pipelines, nnU‐Net automatically configures itself to a given dataset by adapting preprocessing, architecture parameters and training schedules without manual tuning. This is especially valuable for limited datasets like ours, where domain‐specific heuristics can be hard to optimise reliably. Among the available nnU‐Net configurations (2D, 3D low‐res, 3D full‐res and cascade), the 3D full‐resolution model was chosen to fully exploit the 3D spatial context present in the volumetric CT scans and achieve high anatomical accuracy in segmentation. The sample size for training the DL model was chosen based on the work by Huysentruyt et al. [16]. The performance of the DL model was assessed by calculating three commonly used metrics for segmentation: the dice similarity coefficient (DSC), Hausdorff distance (HD) and 95th‐percentile HD (HD95) [37]. These measures are computed for each validation split across the five folds and then averaged over all validation cases. Training of the DL model and inference were performed on a NVIDIA Ampere A100 GPU.

#### Cascaded SSM fitting and landmark transfer

After nnU‐Net segmentation, a landmark transfer methodology was applied to register each patient‐specific bone segmentation to the corresponding SSM bone template. Through this landmark transfer procedure, 36 skeletal landmarks that were predefined on the template were directly transferred to the patient‐specific bone. In addition to the individual landmarks, sets of vertices required for indirect identification were also defined on the template and subsequently automatically transferred through this procedure. These included vertex sets comprising the femoral head centre, femoral neck, proximal and distal femoral shaft and tibial shaft. The remaining nine landmarks were automatically derived indirectly after registration. This step is necessary because nnU‐Net produces voxel‐wise segmentation masks without explicit anatomical correspondences or landmark definitions. To ensure accurate and robust anatomical landmark identification, a fully automated, cascaded SSM‐fitting pipeline was implemented, consisting of global and local SSM fitting followed by a nonrigid refinement step.

1) Global SSM fitting
  In the first step, SSMs of the femur, patella, tibia, fibula, talus and calcaneus were fitted to the DL‐based bone reconstructions. The Ghent lower limb model, used in this study, is the largest articulated SSM of the lower limb previously reported in the literature, including 622 samples in anatomical point‐to‐point correspondence obtained from 311 lower limb CT scans [4]. The SSM represents a shape *S* as a linear combination of the mean shape and principal components derived from principal component analysis (PCA):
$$S=\bar{S}+P\alpha ,$$
  where *S* is the shape vector representing the ordered list of vertex coordinates. $\overset{-}{S}$ defines the corresponding average shape, *P* the matrix of eigenvectors of the covariance matrix $C=\frac{1}{N-1}\overset{N}{\underset{i=1}{\sum }}\left(S_{i}-\bar{S}\right){\left(S_{i}-\bar{S}\right)}^{⊤},$ and α vector of weights. SSM fitting involves optimising the weight vector α to best approximate a given target geometry. The eigenvectors are associated and ranked in decreasing order according to the *t* largest eigenvalues, each representing a mode of shape variation as observed over the training data set. The number *t* of eigenvectors retained is determined such that the cumulative variance represented is 98% of the total variance observed.

2) Local SSM fitting
  Fitting the bone SSMs in the first step provides a robust initialisation by capturing the overall anatomical shape and orientation of each bone. However, global models could lack the resolution needed to accurately represent complex local anatomical variations in clinically relevant regions, such as the femoral condyles and the tibial plateau. Therefore, in a second step, region‐specific proximal and distal femur and tibia SSMs are fitted to these areas individually. This hierarchical fitting strategy enables more precise modelling of local geometry.
  The local models were constructed by selecting the proximal and distal regions on the mean shape template of the femur and tibia. These regions were defined as the top and bottom 20% of vertices along the longitudinal axes of the femur and tibia. The corresponding regions across all 311 training samples from the Ghent lower limb model were extracted and aligned using Generalised Procrustes Analysis to remove positional variance before applying PCA. This process resulted in local SSMs that better capture region‐specific anatomical variability. After global fitting, these local SSMs were automatically fitted to their respective anatomical regions to further refine the surface alignment and enhance the accuracy of the landmark transfer procedure.

3) Final landmark transfer
  Once both global and local SSM fittings were completed, the anatomical landmarks from the reference template were transferred to the patient‐specific segmentation through vertex correspondence. However, due to remaining surface mismatches, particularly in areas of high anatomical variability, a final refinement was performed using a nonrigid ICP algorithm [3]. The nonrigid ICP allowed local surface deformations to further improve alignment between the SSM‐derived shape and the DL‐based segmentation. This step ensured that transferred landmarks closely followed the patient's actual bone geometry. As a result, 36 of the 45 required anatomical landmarks were identified directly, while the remaining 9 were derived indirectly. The indirect landmarks were inferred from the established anatomical correspondence, as these do not lie on the bone surface. All landmarks considered in this study, along with their abbreviations, are listed in Table 1. The centre of the femoral head was determined indirectly by fitting a sphere to the femoral head surface and defining the sphere centre as the femoral head centre. The femoral head region was automatically identified through landmark transfer: femoral head vertices were predefined on the template mesh and subsequently transferred to each patient‐specific segmentation. The femoral head centre was subsequently determined by automatically fitting a sphere to the segmented femoral head surface via least‐squares minimisation of point‐to‐sphere distances. The femoral neck centre was defined as the centroid of the femoral neck region. Similar to the femoral head, the femoral neck region was identified on the template and propagated to the patient‐specific meshes using landmark transfer. For the femoral and tibial shaft landmarks, femoral axis 1 (FA1), femoral axis 2 (FA2), tibial axis 1 (TA1) and tibial axis 2 (TA2) were defined as the midpoints of the proximal and distal shaft regions, respectively. The shaft regions were predefined on the template mesh based on anatomical criteria and transferred to the patient‐specific segmentations via the same landmark transfer procedure. For each shaft region, the midpoint was computed as the centroid of the corresponding set of vertices. This template‐based landmark transfer strategy ensured anatomical correspondence across subjects and allowed for consistent and fully automated landmark extraction across the dataset. The tibial centre proximal (TCP), tibial centre distal (TCD) and talar dome centre (TDC) were automatically identified as the midpoints of tibial lateral condyle centre (TLCC) and tibial medial condyle centre (TMCC), tibial medial malleolus (TMM) and tibial lateral malleolus (TLM) and talar dome lateral (TDL) and talar dome medial (TDM), respectively.

#### Knee and ankle joint coordinate system

**Figure 3:**
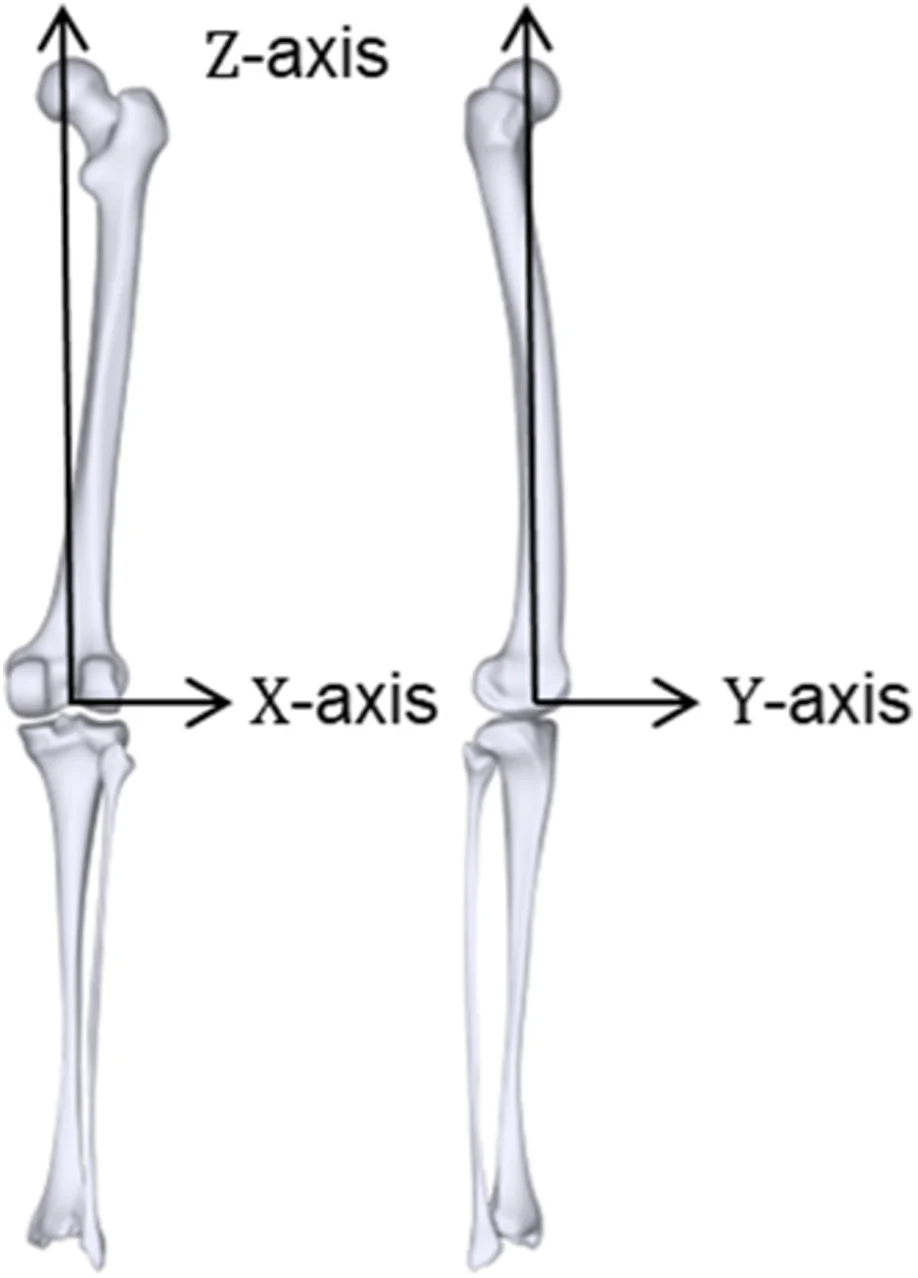
Positioning a right‐sided lower limb in the coordinate system. The *z*‐axis was defined as the mechanical axis of the femoral bone, connecting the centre of the knee with the centre of the femoral head. The *x*‐axis was defined as a parallel of the posterior condylar line (PCL), passing through the centre of the coordinate system, perpendicular to the *z*‐axis. The *y*‐axis was defined as the resultant of the *x*‐ and *z*‐axis based on the right‐hand rule. The coronal plane is defined by the *x*‐ and *z*‐axes, the sagittal plane by the *y*‐ and *z*‐axes, and the axial plane by the *x*‐ and *y*‐axes.

For assessment of the lower limb, we adopted the local coordinate system previously proposed by Victor et al. [41]. The *z*‐axis is defined as the mechanical axis of the femoral bone, running from the centre of the knee to the centre of the femoral head [5, 15]. The centre of the knee is identified as the most posterior point of the trochlear groove (or top of the femoral notch) and serves as the origin in the adapted coordinate system. The *x*‐axis is defined as a parallel line to the posterior condylar line (PCL), which passes through the centre of the coordinate system and perpendicular to the *z*‐axis. The PCL is determined as the line connecting the automatically identified posterior femoral condylar landmarks (femoral lateral condyle posterior [FLCP] and femoral medial condyle posterior [FMCP], Table 1) after the landmark transfer procedure. The *y*‐axis is defined using the right‐hand rule. This completes the three‐dimensional coordinate system (Figure 3). Accordingly, the coronal plane is defined by the *x*‐ and *z*‐axes, the sagittal plane by the *y*‐ and *z*‐axes, and the axial plane by the *x*‐ and *y*‐axes.

For the foot and ankle measurements, an anatomical reference system was defined with the *z*‐axis orthogonal to the ground. The *y*‐axis extends from the most plantar vertex of the calcaneus to the centre of the head of the second metatarsal bone, projected to the ground plane. The centre of the metatarsal head was automatically identified by fitting a sphere to the metatarsal head. The origin was chosen as the centre of the talus. All feet were realigned to this anatomical reference frame as previously discussed in [6].

#### From 3D landmarks to automated measurements

After aligning the 3D bone models to their corresponding anatomical reference frames and automatically identifying the necessary landmarks, the measurements were derived. Landmark pairs defined lines in 3D space. To compute the angular measurements, these 3D lines were projected onto the respective anatomical planes, after which the angles between the projected lines were calculated. Table 2 summarises the landmarks used for each measurement and the corresponding anatomical planes.

**Table 2:** Overview of the 28 orthopaedic measurements. The different landmarks involved in the measurements are defined under Line 1 and Line 2. After projecting the lines between the landmarks onto the anatomical planes, the measurements were calculated. TT‐TG is an exception, as it is the only distance measurement: the linear distance between the tibial tuberosity (TTP) and the trochlear groove (TGCP), projected onto the axial plane perpendicular to the PCL.

Measurement	Acronym	Line 1	Line 2	Plane
Hip‐knee‐ankle angle	HKA	FHC‐COK	COK‐TCD	Coronal
Femoral mechanical angle	FMA	FHC‐COK	FLC‐FMC	
Tibial mechanical angle	TMA	TCP‐TCD	TLCL‐TMCM	
Femoral anatomical angle	FAA	FA1‐FA2	FLC‐FMC	
Tibial anatomical angle	TAA	TA1‐TA2	TLCL‐TMCM	
Femoral mechanical versus anatomical angle	FMvsA	FA1‐FA2	FHC‐COK	
Joint line convergence angle	JLCA	FLC‐FMC	TLCL‐TMCM	
Femoral neck‐shaft angle	FNSA	FHC‐FNC	FA1‐FA2	
Talar tilt	TT	LDTAS‐MDTAS	TDL‐TDM	
Lateral distal tibial angle	LDTA	LDTAS‐MDTAS	TA1‐TA2	
Hindfoot angle	HA	TDC‐CIP	*z*‐axis (foot and ankle frame)	
Anatomical transepicondylar axis versus posterior condylar line	aTEA‐PCL	FME‐FLE	FLCP‐FMCP	Axial
Surgical transepicondylar axis versus posterior condylar line	sTEA‐PCL	FMS‐FLE	FLCP‐FMCP	
Anatomical versus surgical transepicondylar axis angle	asTEA	FME‐FLE	FMS‐FLE	
Femoral anteversion angle	FAVA	FHC‐FNC	FLCP‐FMCP	
Tibial transverse axis versus posterior condylar line	TTA‐PCL	TLCC‐TMCC	FLCP‐FMCP	
Femoral transverse axis versus posterior condylar line	FTA‐PCL	FLCC‐FMCC	FLCP‐FMCP	
Femoral transverse axis versus tibial transverse axis	FTA‐TTA	FLCC‐FMCC	TLCC‐TMCC	
Femoral transverse axis versus trochlear antero‐posterior axis	FTA‐TAPA	FLCC‐FMCC	TGCP‐COK	
Tibial external version angle	TEVA	TLM‐TMM	TPPPL‐TPPPM	
Tibial tuberosity trochlear groove distance	TT‐TG	N/A	N/A	
Sulcus angle	SA	TRLP‐TGCP	TGCP‐TRMP	
Patellar tilt angle	PT	PMP‐PLP	FLCP‐FMCP	
Lateral trochlear inclination	LTI	TRLP‐TGCP	FLCP‐FMCP	
Medial tibial slope	MTS	TMCA‐TMCP	TA1‐TA2	Sagittal
Lateral tibial slope	LTS	TLCA‐TLCP	TA1‐TA2	
Anterior distal tibial angle	ADTA	ADTAS‐PDTAS	TA1‐TA2	
Calcaneal pitch angle	CPA	CIP‐CIPA	*y*‐axis (foot and ankle frame)	

Abbreviations: ADTAS, anterior distal tibial articular surface; CIP, calcaneal inferior point; CIPA, calcaneal inferior point anterior; COK, centre of knee; FA1, femoral axis 1; FA2, femoral axis 2; FHC, femoral head centre; FLC, femoral lateral condyle; FLCC, femoral lateral condyle centre; FLCP, femoral lateral condyle posterior; FLE, femoral lateral epicondyle; FMC, femoral medial condyle; FMCC, femoral medial condyle centre; FMCP, femoral medial condyle posterior; FME, femoral medial epicondyle; FMS, femoral medial sulcus; FNC, femoral neck centre; LDTAS, lateral distal tibial articular surface; MDTAS, medial distal tibial articular surface; PDTAS, posterior distal tibial articular surface; TA1, tibial axis 1; TA2, tibial axis 2; TCD, tibial centre distal; TCP, tibial centre proximal; TDC, talar dome centre; TDL, talar dome lateral; TDM, talar dome medial; TGCP, trochlear groove central point; TLCC, tibial lateral condyle centre; TLCL, tibial lateral condyle lateral; TLM, tibial lateral malleolus; TMCA, tibial medial condyle anterior; TMCC, tibial medial condyle centre; TMCM, tibial medial condyle medial; TMCP, tibial medial condyle posterior; TMM, tibial medial malleolus; TPPPL, tibial posterior proximal plateau lateral; TPPPM, tibial posterior proximal plateau medial; TRLP, trochlear ridge lateral point; TRMP, trochlear ridge medial point.

### Manual 3D landmark identification

The 28 orthopaedic measurements were manually performed by three software‐trained observers on both the WBCT images and consecutively on the DL‐based bone segmentations. One observer repeated the measurements after a 2‐week interval to assess intraobserver variability and minimise memorisation bias. A predefined protocol including the different anatomical landmarks guided the measurements and a dedicated training was provided beforehand to the observers in order to guarantee a standardised measurements protocol. The considered landmarks on the WBCT images were identified in Mimics (Materialise NV) using the scanner‐based reference system defined by the WBCT acquisition. The manual landmark identification on the 3D bone models were completed using 3‐Matic (Materialise NV). In general, the required landmarks were identified on both the WBCT images and the 3D bone models. After projecting the corresponding landmark pairs onto the anatomical planes, the respective angles were calculated. More detailed information regarding the manual measurements on the WBCT data can be found in Appendix A2.

### Statistical analysis

The intraclass correlation coefficient (ICC) for intraobserver differences was calculated using a two‐way mixed‐effects, absolute agreement, single‐rater model. A two‐way random‐effects model was applied for interobserver reliability. According to Koo et al., reliability values were categorised as follows: values below 0.50 indicate poor reliability, values between 0.50 and 0.75 indicate moderate reliability, between 0.75 and 0.90 good reliability, and values exceeding 0.90 indicate excellent reliability [21].

Statistics of the automated measurements were computed across the 30 subjects, providing the average value and standard deviation for the dataset. Additionally, the mean absolute error (MAE) was calculated to assess the accuracy of the automated methodology. This MAE was calculated between the automated measurements and the average of the 3D bone model measurements performed by three manual observers.

## RESULTS

### Automated bone segmentation

The results of the 5‐fold cross‐validation of the DL‐based segmentation of the lower limb bones are listed in Table 3. The average validation DSC exceeded 0.96 for all bones. Furthermore, the average validation HD for all bones consistently measured below 3 mm, with the exception of the femur, which exhibited an average HD of 5.50 ± 2.73 mm. The HD95 was lower than 1 mm on average for all bones.

**Table 3:** Results of the five‐fold cross‐validation of the deep learning‐based bone segmentation. The dice similarity coefficient (DSC), Hausdorff distance (HD) and the 95th‐percentile HD (HD95) are used as evaluation metrics.

	DSC	HD (mm)	HD95 (mm)
Femur	0.98 ± 0.006	5.50 ± 2.73	0.99 ± 0.42
Patella	0.97 ± 0.007	1.81 ± 1.19	0.74 ± 0.40
Tibia	0.98 ± 0.004	2.38 ± 1.23	0.62 ± 0.20
Fibula	0.96 ± 0.009	2.09 ± 1.18	0.58 ± 0.21
Calcaneus	0.99 ± 0.003	2.24 ± 2.00	0.50 ± 0.18
Talus	0.98 ± 0.002	2.80 ± 2.27	0.48 ± 0.15

### Automated measurements

**Figure 4:**
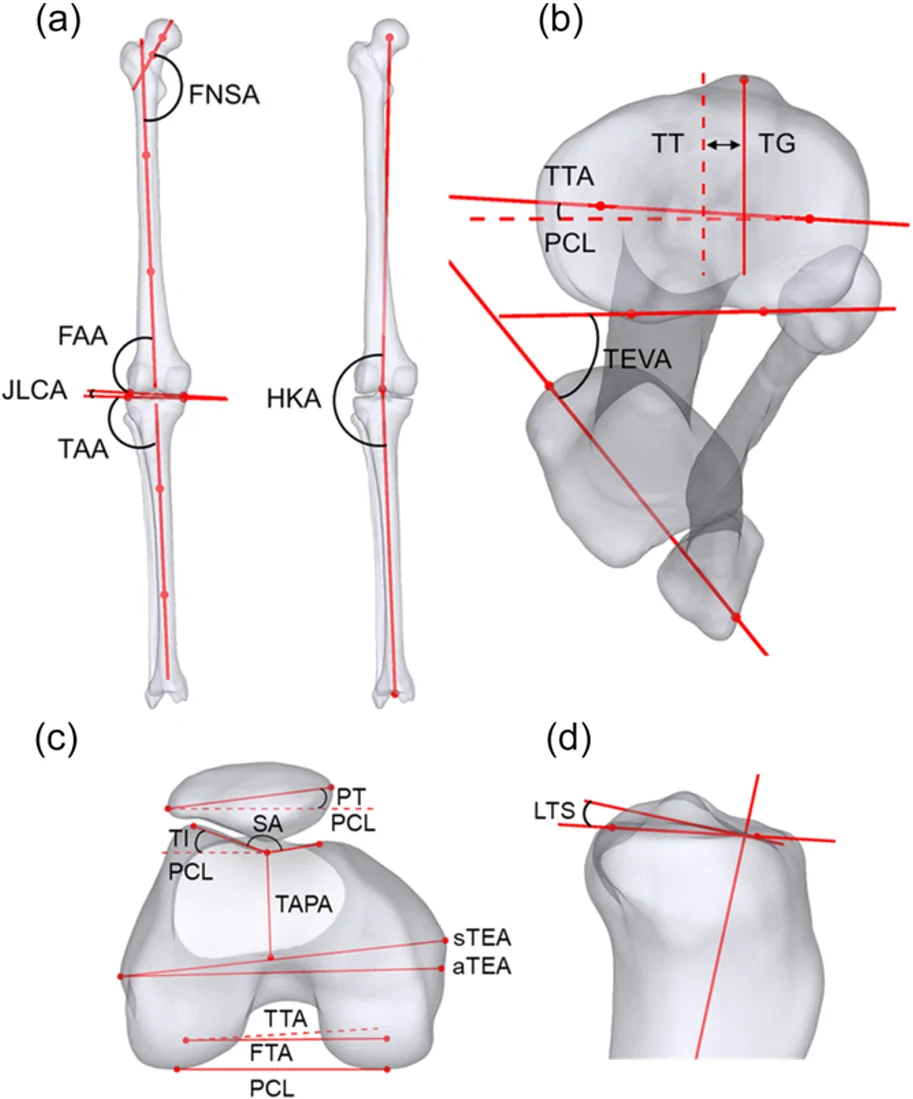
Visualisation of several automated measurements performed on subject 12. (a) depicts HKA, FAA, TAA, JLCA and FNSA on the femur and tibia in the coronal plane; (b) depicts TTA‐PCL, TT‐TG and TEVA measurements on the tibia in the axial plane; (c) depicts the PT, SA, LTI, FTA‐TAPA, sTEA‐PCL, aTEA‐PCL, FTA‐TTA, FTA‐PCL measurements on the distal femur in the axial plane and (d) depicts the LTS in the sagittal plane on proximal tibia. The landmarks required for these measurements can be found in Table 2. aTEA, anatomical transepicondylar axis; FAA, femoral anatomical angle; FNSA, femoral neck‐shaft angle; FTA, femoral transverse axis; HKA, hip‐knee‐ankle angle; JLCA, joint line convergence angle; LTI, lateral trochlear inclination; LTS, lateral tibial slope; PCL, posterior condylar line; PT, patellar tilt; SA, sulcus angle; sTEA, surgical transepicondylar axis; TAA, tibial anatomical angle; TAPA, trochlear antero‐posterior axis; TEVA, tibial external version angle; TT‐TG, tibial tuberosity trochlear groove; TTA, tibial transverse axis.

The mean automated measurements for 30 subjects and the corresponding MAE relative to manual measurements are presented in Table 4. The MAE for the angular measurements ranged from 0.35° ± 0.39° (femoral mechanical versus anatomical angle [FMvsA]) to 5.53° ± 4.68° (sulcus angle [SA]). The MAE for the tibial tuberosity trochlear groove (TT‐TG) measurement was 1.29 mm ± 0.90 mm. The entire pipeline, including DL‐based bone segmentation, SSM fitting and measurement calculation, took an average of 10 min per case. Figure 4 visualises several automated measurements for a specific subject in the respective anatomical planes.

**Table 4:** Summary of the automated measurements with average and standard deviation over the 30 subjects, along with the mean absolute error (MAE) between the automated methodology and the average of the 3D bone model measurements performed by three manual observers.

	Average ± SD	MAE ± SD
HKA	180.00° ± 3.34°	0.55° ± 0.16°
FMA	87.08° ± 1.80°	0.75° ± 0.52°
TMA	92.43° ± 2.44°	0.59° ± 0.38°
FAA	81.84° ± 1.99°	0.55° ± 0.52°
TAA	91.92° ± 2.70°	1.06° ± 0.70°
FMvsA	5.24° ± 1.12°	0.35° ± 0.39°
JLCA	1.01° ± 1.56°	0.78° ± 0.59°
FNSA	131.04° ± 6.56°	3.13° ± 2.60°
FAVA	9.86° ± 9.91°	2.87° ± 2.28°
aTEA‐PCL	5.74° ± 1.28°	1.08° ± 0.69°
sTEA‐PCL	0.93° ± 1.36°	1.08° ± 0.91°
asTEA	4.82° ± 0.18°	0.47° ± 0.31°
TTA‐PCL	6.50° ± 6.26°	1.64° ± 1.23°
FTA‐PCL	1.08° ± 1.26°	1.42° ± 0.90°
FTA‐TTA	7.58° ± 6.13°	2.20° ± 1.61°
FTA‐TAPA	88.59° ± 2.38°	2.29° ± 1.61°
TEVA	37.10° ± 7.59°	1.83° ± 1.87°
TT‐TG	14.26 mm ± 4.71 mm	1.29 mm ± 0.90 mm
PT	13.62° ± 5.94°	2.63° ± 1.69°
LTI	17.70° ± 3.77°	1.95° ± 1.37°
SA	149.28° ± 8.18°	5.53° ± 4.68°
MTS	9.51° ± 2.85°	1.34° ± 1.17°
LTS	7.72° ± 2.51°	1.41° ± 1.18°
LDTA	91.22° ± 2.89°	2.11° ± 1.19°
TT	0.68° ± 1.35°	1.91° ± 1.01°
HA	1.43° ± 5.55°	0.48° ± 0.51°
ADTA	83.77° ± 2.52°	0.77° ± 0.57°
CPA	24.39° ± 6.57°	0.69° ± 0.58°

Abbreviations: ADTA, anterior distal tibial angle; aTEA, anatomical transepicondylar axis; CPA, calcaneal pitch angle; FAA, femoral anatomical angle; FAVA, femoral anteversion angle; FMA, femoral mechanical angle; FMvsA, femoral mechanical versus anatomical angle; FNSA, femoral neck‐shaft angle; FTA, femoral transverse axis; HA, hindfoot angle; HKA, hip‐knee‐ankle angle; JLCA, joint line convergence angle; LDTA, lateral distal tibial angle; LTI, lateral trochlear inclination; LTS, lateral tibial slope; MTS, medial tibial slope; PCL, posterior condylar line; PT, patellar tilt; SA, sulcus angle; SD, standard deviation; sTEA, surgical transepicondylar axis; TAA, tibial anatomical angle; TAPA, trochlear antero‐posterior axis; TEVA, tibial external version angle; TMA, tibial mechanical angle; TT, talar tilt; TTA, tibial transverse axis; TT‐TG, tibial tuberosity trochlear groove.

### Reliability assessment

The results of the ICC analysis for the 28 different measurements are presented in Appendix Supplemental Table 1. Averaging the pairwise ICC values for manual measurements performed on the 3D bone models vs automated measurements showed good to excellent reliability for 18 measurements, moderate reliability for 5 measurements (FTA‐TAPA, JLCA, LTS, SA, LTI) and poor reliability for the 5 remaining measurements (asTEA, aTEA‐PCL, sTEA‐PCL, FTA‐PCL, TT).

Averaging the pairwise ICC values for manual measurements performed directly on the WBCT images vs automated measurements showed good to excellent reliability for 12 measurements, moderate reliability for 6 measurements (FMA, FAA, JLCA, FAVA, LTI, LTS), and poor reliability for the remaining 10 measurements (FMvsA, aTEA‐PCL, sTEA‐PCL, asTEA, FTA‐PCL, FTA‐TAPA, SA, LDTA, TT, ADTA).

Comparison of interobserver ICC values between manual measurements performed on WBCT images and the manual measurements performed on 3D bone models revealed that 18 out of 28 measurements exhibited lower interobserver reliability when measured on WBCT images. These measurements included HKA, FMA, TMA, FAA, TAA, FMvsA, JLCA, FNSA, FAVA, TTA‐PCL, FTA‐TTA, TT‐TG, SA, MTS, LDTA, HA, ADTA and CPA.

## DISCUSSION

In this study, a hybrid DL‐SSM methodology was developed for fully automated 3D lower limb landmarking directly from full‐leg WBCT imaging data. The proposed methodology combines DL‐based bone segmentation with SSMs to incorporate prior anatomical constraints during landmark transfer. From these automatically identified landmarks, 28 clinically relevant measurements describing lower limb alignment and morphology were derived. We found that our automated method corresponded well to manual assessments, with differences that generally matched the interobserver reliability of the manual method.

The accuracy of these automated measurements depends on the quality of the underlying bone segmentations. As a first step, the performance of DL‐based segmentation model was assessed on full‐leg WBCT data. To the best of our knowledge, this is the first study to develop an nnU‐Net‐based automated segmentation methodology for full‐leg WBCT imaging. When comparing the WBCT segmentation results to previously published work on conventional CT, the achieved accuracy is comparable in terms of DSC. Kuiper et al. reported a mean DSC value of 0.98 across the lower limb bones [22]. In this study, using the WBCT dataset, we obtained similar DSC values, from 0.96 ± 0.009 for the fibula to 0.99 ± 0.003 for the calcaneus. The corresponding HD values in this study, ranging from 1.8 to 5.5 mm, are in line with the available literature on automated lower limb segmentation [2, 22, 23]. The highest HD was observed for the femur (5.5 mm ± 2.7 mm), which is slightly higher than those reported by Kuiper et al. (3.5 mm ± 5.5 mm and 5.0 mm ± 3.2 mm, depending on the dataset). In contrast, HD values for the calcaneus, talus and tibia were lower than those reported in the same study. The higher HD values observed for the femur can be attributed to the characteristics of WBCT imaging, which is more prone to image noise, scatter and patient motion artifacts than conventional supine CT [8, 40]. Moreover, the HD is a strict metric that reflects the largest point‐wise deviation between surfaces and is therefore sensitive to local outliers. Therefore, the HD95 was also evaluated. On average, all HD95 values were below 1 mm, demonstrating the capability of DL‐based methods for accurate and reliable lower limb bone segmentation from WBCT data.

From the ICC analysis comparing the automated method to manual measurements performed on the 3D bone models by the observers, five measurements (FTA‐TAPA, JLCA, LTS, SA and LTI) were found to have moderate reliability, and five measurements (asTEA, aTEA‐PCL, sTEA‐PCL, FTA‐PCL and TT) showed poor reliability. This is comparable to the interobserver reliability among the three human raters on the 3D bone models, where four measurements (FTA–TAPA, JLCA, aTEA–PCL and LTS) exhibited moderate reliability, and six (SA, LTI, sTEA–PCL, asTEA, FTA–PCL and TT) demonstrated poor reliability. This indicates that lower ICC values between the automated and manual measurements were partly due to variability already present among human observers, making it challenging to achieve high agreement for these particular measurements. Kuiper et al. reported good to excellent ICC values for 23 out of 25 measurements when comparing manual and automated methods. While their study established a ground truth based on a single rater's manual landmark annotation on 3D bone models, we refined this methodology by deriving a more robust ground truth from the average of three independent raters' manual segmentations. Furthermore, we extended the scope of the comparisons by including manual measurements on both 3D models and WBCT scans. The observed moderate to poor interobserver reliability for several angles, including SA, asTEA, aTEA‐PCL, FTA‐TAPA and FTA‐PCL, was consistent with findings from other studies evaluating the precision of manual landmark selection and measurements [18, 36, 42]. Although these specific patellofemoral measurements were not included in the study by Kuiper et al., they are critical for assessing patellofemoral instability and patellofemoral kinematics [23]. While landmark picking and angular measurement reliability are not directly equivalent, measurement repeatability inherently depends on the precision of the underlying landmark positions. Compounded effects can occur when landmarks with lower reliability are involved in angle calculations. When comparing the ICC values of measurements derived from 3D bone models and from WBCT images, interobserver reliability was generally higher for measurements performed on the 3D bone models. Manual identification of anatomical landmarks on coronal, sagittal and axial CT image slices can be more challenging and prone to variability. In contrast, the use of reconstructed 3D bone models provides a more comprehensive anatomical overview, which may facilitate landmark identification and result in higher interobserver reliability.

Orthopaedic practice still generally relies on 2D radiographic imaging and measurements. While 2D x‐rays are practical and widely available, they project 3D anatomy onto a 2D plane, causing loss of spatial information and projection or rotational bias [7, 29]. Advanced 3D imaging, such as WBCT, provides anatomically accurate representations under physiological loading but is more complex and time‐consuming to analyse. Jud et al. highlighted the importance of developing 3D planning methods that incorporate weight‐bearing conditions, as significant differences are observed in alignment parameters such as the HKA angle and JLCA between weight‐bearing and non‐weight‐bearing imaging [19]. Because bone morphology is inherently three‐dimensional, it should ideally be performed directly on 3D bone models, where they are free from projection and rotation bias. Several recent studies support this shift towards 3D‐based analysis, which better captures true alignment and morphology [10, 14, 19, 33, 38]. However, translating 2D definitions into 3D is challenging, as landmark identification becomes less standardised and few established 3D measurement protocols exist. The results in this work reflect these limitations: despite the advantages of 3D imaging, interobserver variability persists due to the subjective nature of manual landmark selection, consistent with previous findings by Victor et al. and van der Merwe et al. [27, 42]. This variability has clinical relevance, as inconsistent measurements may lead to different diagnostic or therapeutic decisions [20]. Looking forward, we believe that automated 3D image analysis represents an important step towards overcoming these limitations. By standardising landmark detection and measurement computation, automated systems can enhance the reliability, reproducibility and efficiency of 3D assessments, ultimately supporting more consistent and objective evaluations of lower limb alignment and morphology.

There were several limitations associated with the present study. A primary limitation is that the dataset predominantly comprised patients without major anatomical deformities, implants or advanced pathologies, which may influence the accuracy of both manual and automated measurements. For instance, the presence of orthopaedic implants could interfere with automated segmentation, as the DL model was not trained on such cases. Similarly, severe osteoarthritis or other pathological changes could impact model performance in two critical ways: first, bone deformities may compromise segmentation accuracy; second, the SSM fitting process could be affected, as such anatomical variability was not represented in the training set used to build the SSM. Consequently, landmark transfer in these cases could be suboptimal, resulting in inaccurate or unreliable measurements. A second limitation is the relatively small sample size of 30 patients. Although initial results demonstrate the potential of the proposed methodology, future work should include validation on a larger and more heterogeneous dataset to further substantiate its generalisability and clinical applicability. A third limitation is that intraobserver reliability of the manual measurements was only partially assessed, based on a single observer performing one nonrandomised repetition. A final limitation of this study is the use of a femur‐based anatomical reference frame for measurements that include the tibia and fibula, which does not explicitly account for flexion or rotation of the lower leg relative to the femur. However, patients were instructed to stand with fully extended knees and parallel feet during image acquisition.

In conclusion, leveraging deep learning with statistical shape models demonstrates potential for automating lower limb alignment and morphology assessment using weight‐bearing CT imaging data. Automated landmark identification provides consistent measurements, standardises assessments and reduces observer bias and workload, resulting in a more robust and reproducible measurement approach. Furthermore, our study demonstrated that performing manual measurements on 3D bone models reduces observer variability compared to measurements on CT scans, as evidenced by the improved ICCs among raters.

## AUTHOR CONTRIBUTIONS

**Ide Van den Borre**: Writing—review and editing; writing—original draft; visualisation; validation; supervision; software; methodology; data curation. **Emmanuel Audenaert**: Conceptualisation; data curation; funding acquisition; methodology; software; supervision; writing—review and editing. **Hannes Vermue**: Conceptualisation; writing—review and editing. **Roel Huysentruyt**: Conceptualisation; software; writing—review and editing. **Leonie Van Vynckt**: Data curation; writing—review and editing. **Robin Vanhauwe**: Data curation; writing—review and editing. **Victor Pas**: Data curation; writing—review and editing; **Aleksandra Pizurica**: Funding acquisition; supervision; data curation; writing—review and editing. **Aline Van Oevelen**: Writing—review and editing; visualisation; validation; supervision; methodology; software; data curation.

## CONFLICT OF INTEREST STATEMENT

The authors declare that they have no conflicts of interest related to this work.

## ETHICS STATEMENT

The study B6702022000639 was approved by the Commissie voor Medische Ethiek (Medical Ethics Committee) of Ghent University and Ghent University Hospital (UZ Gent). Informed consent was obtained from all individual participants included in the study.

## Supporting information

Appendix.

## Data Availability

Research data are not shared.
