# Supplementary material for: Combining deep learning with statistical shape modelling enables automated lower limb measurements with observer‐level reliability using weight‐bearing computed tomography
Source: J Exp Orthop. 2026 Feb 26;13(1):e70669. doi: 10.1002/jeo2.70669 (PMC12936986; doi:10.1002/jeo2.70669)
Supplement: Supplementary file 1 — Appendix. [file JEO2-13-e70669-s001.docx]

**Appendix**

## **A1. Tables**

Table 1: Results of the ICC analysis between the manual observers and the automated method. Intraobserver reliability was assessed for observer 2, who performed the same measurements twice.

|  | CT | | | 3D bone model | | |
| --- | --- | --- | --- | --- | --- | --- |
| Measurement | Observer 2 | Observer 3 | Automated | Observer 2 | Observer 3 | Automated |
| HKA |  |  |  |  |  |  |
| Observer 1 | 0.99 (0.98, 1.0) | 0.98 (0.95, 0.99) | 0.87 (0.75, 0.94) | 1.00 (0.95, 1.00) | 1.00 (0.97, 1.00) | 1.00 (1.00, 1.00) |
| Observer 2 | 0.99 (0.99, 1.00) | 0.98 (0.95, 0.99) | 0.88 (0.77, 0.94) | 1.00 (1.00, 1.00) | 1.00 (1.00, 1.00) | 1.00 (1.00, 1.00) |
| Observer 3 |  |  | 0.88 (0.76, 0.94) |  |  | 1.00 (1.00, 1.00) |
| FMA |  |  |  |  |  |  |
| Observer 1 | 0.69 (0.23, 0.87) | 0.79 (0.61, 0.9) | 0.66 (0.39, 0.82) | 0.98 (0.95, 0.99) | 0.97 (0.94, 0.99) | 0.91 (0.82, 0.96) |
| Observer 2 | 0.89 (0.79, 0.95) | 0.69 (0.36, 0.86) | 0.74 (0.51, 0.87) | 0.97 (0.93, 0.98) | 0.98 (0.97, 0.99) | 0.92 (0.83, 0.96) |
| Observer 3 |  |  | 0.68 (0.43, 0.84) |  |  | 0.94 (0.88, 0.97) |
| TMA |  |  |  |  |  |  |
| Observer 1 | 0.8 (0.63, 0.9) | 0.87 (0.73, 0.93) | 0.84 (0.68, 0.92) | 0.90 (0.80, 0.95) | 0.95 (0.90, 0.98) | 0.96 (0.93, 0.98) |
| Observer 2 | 0.71 (0.47, 0.85) | 0.85 (0.70, 0.92) | 0.82 (0.65, 0.91) | 0.92 (0.84, 0.96) | 0.85 (0.70, 0.93) | 0.94 (0.87, 0.97) |
| Observer 3 |  |  | 0.81 (0.64, 0.91) |  |  | 0.89 (0.77, 0.94) |
| FAA |  |  |  |  |  |  |
| Observer 1 | 0.65 (0.11, 0.86) | 0.66 (0.38, 0.82) | 0.60 (0.31, 0.79) | 0.97 (0.92, 0.99) | 0.93 (0.86, 0.97) | 0.92 (0.83, 0.96) |
| Observer 2 | 0.88 (0.76, 0.94) | 0.67 (0.41, 0.83) | 0.62 (0.33, 0.80) | 0.94 (0.88, 0.97) | 0.96 (0.93, 0.98) | 0.91 (0.82, 0.96) |
| Observer 3 |  |  | 0.80 (0.61, 0.90) |  |  | 0.95 (0.89, 0.97) |
| TAA |  |  |  |  |  |  |
| Observer 1 | 0.84 (0.69, 0.92) | 0.85 (0.70, 0.93) | 0.81 (0.63, 0.91) | 0.91 (0.83, 0.96) | 0.94 (0.88, 0.97) | 0.96 (0.91, 0.98) |
| Observer 2 | 0.94 (0.87, 0.97) | 0.81 (0.45, 0.92) | 0.77 (0.56, 0.88) | 0.91 (0.81, 0.95) | 0.90 (0.81, 0.95) | 0.93 (0.86, 0.97) |
| Observer 3 |  |  | 0.77 (0.57, 0.89) |  |  | 0.92 (0.83, 0.96) |
| FMvsA |  |  |  |  |  |  |
| Observer 1 | 0.79 (0.59, 0.9) | 0.56 (0.13, 0.79) | 0.28 (-0.09, 0.58) | 0.96 (0.90, 0.98) | 0.89 (0.78, 0.95) | 0.94 (0.87, 0.97) |
| Observer 2 | 0.81 (0.63, 0.91) | 0.68 (0.35, 0.85) | 0.42 (0.07, 0.68) | 0.92 (0.84, 0.96) | 0.90 (0.81, 0.95) | 0.82 (0.66, 0.91) |
| Observer 3 |  |  | 0.67 (0.40, 0.83) |  |  | 0.92 (0.83, 0.96) |
| JLCA |  |  |  |  |  |  |
| Observer 1 | 0.62 (0.13, 0.83) | 0.68 (0.43, 0.84) | 0.57 (0.27, 0.77) | 0.76 (0.56, 0.88) | 0.75 (0.54, 0.87) | 0.80 (0.62, 0.90) |
| Observer 2 | 0.94 (0.88, 0.97) | 0.49 (0.08, 0.75) | 0.43 (0.08, 0.68) | 0.79 (0.60, 0.89) | 0.65 (0.40, 0.82) | 0.73 (0.50, 0.86) |
| Observer 3 |  |  | 0.72 (0.49, 0.86) |  |  | 0.63 (0.35, 0.80) |
| FNSA |  |  |  |  |  |  |
| Observer 1 | 0.74 (0.53, 0.87) | 0.73 (0.50, 0.87) | 0.74 (0.53, 0.87) | 0.88 (0.77, 0.94) | 0.89 (0.76, 0.95) | 0.81 (0.64, 0.91) |
| Observer 2 | 0.97 (0.93, 0.98) | 0.89 (0.78, 0.95) | 0.87 (0.74, 0.94) | 0.80 (0.63, 0.90) | 0.86 (0.71, 0.93) | 0.79 (0.61, 0.90) |
| Observer 3 |  |  | 0.83 (0.67, 0.92) |  |  | 0.89 (0.78, 0.95) |
| FAVA |  |  |  |  |  |  |
| Observer 1 | 0.71 (0.44, 0.86) | 0.52 (0.20, 0.74) | 0.57 (0.26, 0.77) | 0.94 (0.88, 0.97) | 0.81 (0.61, 0.91) | 0.94 (0.89, 0.97) |
| Observer 2 | 0.89 (0.78, 0.95) | 0.73 (0.51, 0.87) | 0.84 (0.68, 0.92) | 0.73 (0.50, 0.86) | 0.85 (0.69, 0.93) | 0.80 (0.62, 0.90) |
| Observer 3 |  |  | 0.66 (0.39, 0.82) |  |  | 0.96 (0.91, 0.98) |
| aTEA-PCL |  |  |  |  |  |  |
| Observer 1 | 0.67 (0.41, 0.83) | 0.74 (0.51, 0.87) | 0.40 (0.05, 0.67) | 0.63 (0.35, 0.81) | 0.53 (0.23, 0.74) | 0.44 (0.10, 0.69) |
| Observer 2 | 0.71 (0.46, 0.85) | 0.69 (0.41, 0.85) | 0.34 (-0.02, 0.63) | 0.47 (0.13, 0.70) | 0.36 (0.01, 0.63) | 0.25 (-0.12, 0.55) |
| Observer 3 |  |  | 0.60 (0.30, 0.79) |  |  | 0.45 (0.11, 0.69) |
| sTEA-PCL |  |  |  |  |  |  |
| Observer 1 | 0.50 (0.14, 0.74) | 0.70 (0.29, 0.87) | 0.57 (0.26, 0.77) | 0.70 (0.35, 0.81) | 0.41 (-0.05, 0.71) | 0.29 (-0.07, 0.58) |
| Observer 2 | 0.67 (0.41, 0.83) | 0.71 (0.46, 0.85) | 0.29 (-0.08, 0.59) | 0.26 (-0.10, 0.56) | 0.36 (-0.05, 0.66) | 0.30 (-0.06, 0.59) |
| Observer 3 |  |  | 0.55 (0.23, 0.76) |  |  | 0.47 (0.14, 0.71) |
| asTEA |  |  |  |  |  |  |
| Observer 1 | 0.06 (-0.20, 0.36) | 0.02 (-0.10, 0.22) | 0.00 (-0.36, 0.36) | 0.29 (-0.08, 0.59) | 0.01 (-0.20, 0.28) | 0.05 (-0.31, 0.40) |
| Observer 2 | 0.32 (-0.04, 0.61) | 0.18 (-0.14, 0.49) | -0.05 (-0.40, 0.32) | 0.01 (-0.35, 0.36) | -0.13 (-0.37, 0.17) | 0.12 (-0.25, 0.46) |
| Observer 3 |  |  | -0.16 (-0.49, 0.21) |  |  | -0.15 (-0.48, 0.22) |
| TTA-PCL |  |  |  |  |  |  |
| Observer 1 | 0.64 (0.26, 0.83) | 0.75 (0.53, 0.87) | 0.75 (0.53, 0.87) | 0.68 (0.42, 0.83) | 0.65 (0.36, 0.82) | 0.78 (0.59, 0.89) |
| Observer 2 | 0.88 (0.76, 0.94) | 0.71 (0.40, 0.86) | 0.80 (0.61, 0.90) | 0.88 (0.77, 0.94) | 0.84 (0.49, 0.94) | 0.91 (0.83, 0.96) |
| Observer 3 |  |  | 0.75 (0.54, 0.87) |  |  | 0.90 (0.81, 0.95) |
| FTA-PCL |  |  |  |  |  |  |
| Observer 1 | 0.03 (-0.26, 0.36) | 0.08 (-0.21, 0.39) | 0.28 (-0.09, 0.58) | -0.10 (-0.43, 0.26) | -0.03 (-0.40, 0.33) | 0.20 (-0.17, 0.52) |
| Observer 2 | 0.22 (-0.16, 0.54) | 0.39 (0.02, 0.66) | 0.01 (-0.35, 0.37) | -0.07 (-0.42, 0.29) | 0.16 (-0.21, 0.49) | 0.32 (-0.04, 0.60) |
| Observer 3 |  |  | -0.18 (-0.51, 0.20) |  |  | 0.19 (-0.18, 0.51) |
| FTA-TTA |  |  |  |  |  |  |
| Observer 1 | 0.70 (0.46, 0.85) | 0.78 (0.59, 0.89) | 0.86 (0.72, 0.93) | 0.67 (0.42, 0.83) | 0.58 (0.29, 0.77) | 0.79 (0.60, 0.89) |
| Observer 2 | 0.82 (0.66, 0.91) | 0.48 (0.15, 0.71) | 0.83 (0.68, 0.92) | 0.75 (0.53, 0.87) | 0.74 (0.42, 0.88) | 0.82 (0.65, 0.91) |
| Observer 3 |  |  | 0.75 (0.53, 0.87) |  |  | 0.87 (0.75, 0.94) |
| FTA-TAPA |  |  |  |  |  |  |
| Observer 1 | 0.64 (0.37, 0.82) | 0.52 (0.19, 0.74) | 0.11 (-0.26, 0.45) | 0.45 (0.11, 0.70) | 0.45 (0.12, 0.70) | 0.47 (0.14, 0.71) |
| Observer 2 | 0.86 (0.72, 0.93) | 0.70 (0.46, 0.85) | 0.40 (0.05, 0.67) | 0.56 (0.26, 0.77) | 0.60 (0.31, 0.78) | 0.53 (0.22, 0.74) |
| Observer 3 |  |  | 0.54 (0.22, 0.76) |  |  | 0.55 (0.24, 0.76) |
| TEVA |  |  |  |  |  |  |
| Observer 1 | 0.88 (0.49, 0.96) | 0.97 (0.94, 0.99) | 0.89 (0.77, 0.94) | 0.74 (0.09, 0.91) | 0.89 (0.75, 0.95) | 0.94 (0.88, 0.97) |
| Observer 2 | 0.96 (0.91, 0.98) | 0.89 (0.63, 0.96) | 0.86 (0.73, 0.93) | 0.52 (0.21, 0.74) | 0.82 (0.49, 0.93) | 0.91 (0.83, 0.96) |
| Observer 3 |  |  | 0.89 (0.78, 0.95) |  |  | 0.87 (0.74, 0.94) |
| TT-TG |  |  |  |  |  |  |
| Observer 1 | 0.89 (0.77, 0.95) | 0.61 (0.17, 0.82) | 0.74 (0.51, 0.87) | 0.74 (0.53, 0.87) | 0.84 (0.34, 0.94) | 0.93 (0.85, 0.96) |
| Observer 2 | 0.89 (0.79, 0.95) | 0.64 (0.19, 0.84) | 0.79 (0.61, 0.90) | 0.94 (0.88, 0.97) | 0.66 (0.32, 0.84) | 0.89 (0.78, 0.95) |
| Observer 3 |  |  | 0.86 (0.73, 0.93) |  |  | 0.82 (0.65, 0.91) |
| PT |  |  |  |  |  |  |
| Observer 1 | 0.95 (0.89, 0.97) | 0.92 (0.77, 0.97) | 0.95 (0.90, 0.98) | 0.79 (0.61, 0.89) | 0.70 (0.15, 0.88) | 0.94 (0.88, 0.97) |
| Observer 2 | 0.95 (0.91, 0.98) | 0.89 (0.62, 0.96) | 0.93 (0.85, 0.96) | 0.88 (0.76, 0.94) | 0.42 (0.03, 0.69) | 0.76 (0.56, 0.88) |
| Observer 3 |  |  | 0.91 (0.82, 0.96) |  |  | 0.86 (0.73, 0.93) |
| LTI |  |  |  |  |  |  |
| Observer 1 | 0.49 (0.1, 0.74) | 0.62 (0.34, 0.8) | 0.58 (0.27, 0.78) | 0.55 (0.26, 0.76) | 0.40 (-0.03, 0.69) | 0.67 (0.41, 0.83) |
| Observer 2 | 0.84 (0.69, 0.92) | 0.35 (0.02, 0.62) | 0.34 (-0.03, 0.62) | 0.59 (0.30, 0.78) | 0.31 (-0.11, 0.65) | 0.67 (0.41, 0.83) |
| Observer 3 |  |  | 0.60 (0.31, 0.79) |  |  | 0.86 (0.72, 0.93) |
| SA |  |  |  |  |  |  |
| Observer 1 | 0.31 (-0.05, 0.6) | 0.33 (-0.05, 0.62) | 0.26 (-0.11, 0.57) | 0.38 (0.05, 0.64) | 0.45 (0.12, 0.69) | 0.61 (0.33, 0.80) |
| Observer 2 | 0.90 (0.79, 0.95) | 0.06 (-0.15, 0.32) | 0.32 (-0.05, 0.61) | 0.66 (0.40, 0.82) | 0.48 (0.05, 0.74) | 0.69 (0.45, 0.84) |
| Observer 3 |  |  | 0.24 (-0.13, 0.55) |  |  | 0.70 (0.45, 0.84) |
| MTS |  |  |  |  |  |  |
| Observer 1 | 0.87 (0.74, 0.93) | 0.90 (0.61, 0.97) | 0.78 (0.58, 0.89) | 0.84 (0.70, 0.92) | 0.90 (0.80, 0.95) | 0.86 (0.72, 0.93) |
| Observer 2 | 0.92 (0.84, 0.96) | 0.79 (0.45, 0.91) | 0.83 (0.66, 0.91) | 0.88 (0.76, 0.94) | 0.81 (0.64, 0.91) | 0.84 (0.70, 0.92) |
| Observer 3 |  |  | 0.78 (0.58, 0.89) |  |  | 0.78 (0.60, 0.89) |
| LTS |  |  |  |  |  |  |
| Observer 1 | 0.81 (0.64, 0.91) | 0.86 (0.72, 0.93) | 0.57 (0.27, 0.77) | 0.53 (0.21, 0.74) | 0.51 (0.19, 0.74) | 0.75 (0.54, 0.87) |
| Observer 2 | 0.82 (0.66, 0.91) | 0.79 (0.60, 0.89) | 0.55 (0.23, 0.76) | 0.66 (0.39, 0.82) | 0.41 (0.06, 0.67) | 0.54 (0.23, 0.75) |
| Observer 3 |  |  | 0.55 (0.23, 0.76) |  |  | 0.51 (0.18, 0.73) |
| LDTA |  |  |  |  |  |  |
| Observer 1 | 0.33 (-0.05, 0.63) | 0.45 (0.08, 0.71) | 0.23 (-0.15, 0.54) | 0.55 (0.24, 0.76) | 0.56 (0.05, 0.80) | 0.86 (0.72, 0.93) |
| Observer 2 | 0.16 (-0.21, 0.49) | 0.16 (-0.10, 0.44) | 0.42 (0.08, 0.68) | 0.78 (0.59, 0.89) | 0.55 (0.15, 0.78) | 0.76 (0.56, 0.88) |
| Observer 3 |  |  | 0.14 (-0.23, 0.48) |  |  | 0.72 (0.50, 0.86) |
| ADTA |  |  |  |  |  |  |
| Observer 1 | 0.62 (0.21, 0.82) | 0.48 (0.09, 0.73) | 0.08 (-0.29, 0.43) | 0.89 (0.78, 0.95) | 0.62 (0.08, 0.84) | 0.93 (0.85, 0.96) |
| Observer 2 | 0.73 (0.50, 0.86) | 0.66 (0.39, 0.82) | 0.25 (-0.12, 0.56) | 0.55 (0.24, 0.76) | 0.57 (0.01, 0.82) | 0.74 (0.52, 0.87) |
| Observer 3 |  |  | 0.30 (-0.07, 0.60) |  |  | 0.93 (0.86, 0.97) |
| TT |  |  |  |  |  |  |
| Observer 1 | 0.44 (0.11, 0.69) | 0.04 (-0.2, 0.32) | 0.28 (-0.09, 0.59) | 0.14 (-0.23, 0.47) | -0.01 (-0.24, 0.27) | 0.30 (-0.06, 0.59) |
| Observer 2 | 0.42 (0.07, 0.68) | 0.11 (-0.11, 0.38) | 0.37 (0.01, 0.64) | -0.05 (-0.40, 0.31) | 0.26 (-0.05, 0.55) | 0.35 (-0.00, 0.63) |
| Observer 3 |  |  | 0.29 (-0.08, 0.59) |  |  | 0.46 (0.13, 0.70) |
| HA |  |  |  |  |  |  |
| Observer 1 | 0.93 (0.85, 0.97) | 0.86 (0.72, 0.93) | 0.90 (0.80, 0.95) | 0.98 (0.96, 0.99) | 0.98 (0.96, 0.99) | 0.99 (0.99, 1.00) |
| Observer 2 | 1.00 (0.99, 1.00) | 0.87 (0.73, 0.93) | 0.89 (0.77, 0.94) | 0.98 (0.96, 0.99) | 0.99 (0.97, 0.99) | 0.98 (0.96, 0.99) |
| Observer 3 |  |  | 0.81 (0.64, 0.91) |  |  | 0.99 (0.97, 0.99) |
| CPA |  |  |  |  |  |  |
| Observer 1 | 0.93 (0.85, 0.97) | 0.90 (0.64, 0.96) | 0.85 (0.70, 0.93) | 0.96 (0.91, 0.98) | 0.95 (0.90, 0.98) | 0.97 (0.94, 0.99) |
| Observer 2 | 0.92 (0.84, 0.96) | 0.90 (0.76, 0.96) | 0.83 (0.67, 0.92) | 0.98 (0.96, 0.99) | 0.99 (0.97, 0.99) | 0.99 (0.97, 0.99) |
| Observer 3 |  |  | 0.83 (0.67, 0.92) |  |  | 0.99 (0.98, 0.99) |

## **A2. Manual WBCT measurements**

**Overview**

A total of 28 orthopedic measurements were manually performed by three trained observers directly on the WBCT images using Mimics (Materialise NV, Leuven, Belgium). Each measurement was obtained by identifying the necessary anatomical landmarks on the CT images) using the scanner-based reference system defined by the WBCT acquisition. Some landmarks could be directly localized on the cortical bone surface, while others were defined using geometric operations, such as sphere fitting for determination of the femoral head center. For each measurement, 3D landmarks were placed on the relevant slices by scrolling through the volume. Once all landmarks were defined, lines were created between corresponding point pairs and projected onto the appropriate anatomical plane of the WBCT dataset. The angle between these projected lines was then calculated within Mimics. Below we discuss the procedure for the different measurements. An example of the manual WBCT measurement is visualized in Figure A1.


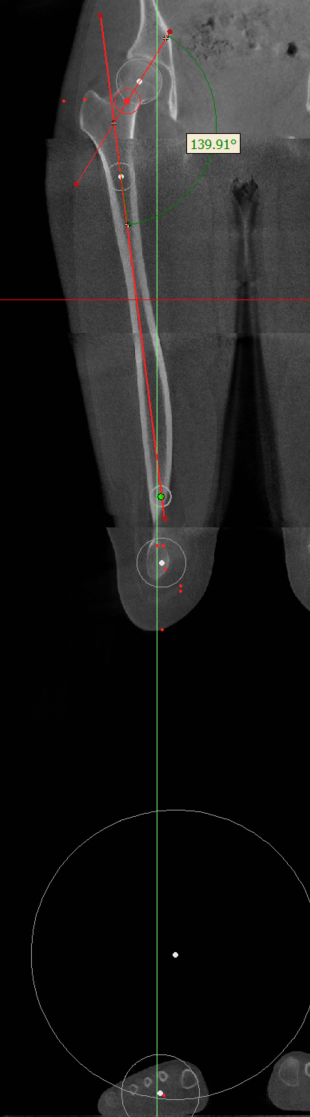


Figure A1: Example of a manual measurement of the FNSA angle on WBCT. Note that the landmarks required to calculate this angle are not necessarily located on the same coronal slice. After identifying the 3D landmarks and the lines connecting them, these lines are projected onto the coronal plane before calculating the angle.

**Coronal plane measurements**

1. HKA

Line from the center of the femoral head to the center of the knee, and from the center of the knee to the center of the ankle.

- Hip center: best-fit sphere to the femoral head
- Knee center: deepest point of the femoral notch.
- Ankle center: midpoint between medial and lateral malleolus.
- Measured laterally.

1. FMA

Angle between femoral mechanical axis and line tangent to distal femoral bone.

- Femoral mechanical axis: connecting hip center with knee center.
- Tangent distal femoral bone: connecting most distal points of the lateral and medial femoral condyles.
- Measured laterally.

1. TMA

Angle between tibial mechanical axis and line tangent to proximal tibial bone.

- Tibial mechanical axis: Connecting center of tibial plateau with center of the distal tibial joint surface.
- Tangent proximal tibial bone: connecting most proximal points on the medial and lateral tibial plateau.
- Measured laterally.

1. FAA

Angle between the femoral anatomical axis and the tangent to the distal femoral condyles.

- Femoral anatomical axis: line through the centers of two best-fitting circles along the femoral shaft (proximal and distal regions).
- Distal femoral tangent: see FMA.
- Measured laterally.

1. TAA

Angle between the tibial anatomical axis and the tangent to the proximal tibial plateau.

- Tibial anatomical axis: line through the centers of two best-fitting circles along the tibial shaft (proximal and distal).
- Proximal tibial tangent: as defined in TMA.
- Measured laterally.

1. FMvA

Angle between the femoral mechanical axis and the femoral anatomical axis, reflecting the difference between mechanical and anatomical alignment of the femur.

- Femoral mechanical axis: see FMA.
- Femoral anatomical axis: see FAA.
- Measured proximally

1. JLCA

Angle between the distal femoral joint line and the proximal tibial joint line.

- Distal femoral tangent: as defined in FMA.
- Proximal tibial tangent: as defined in TMA.
- Measured laterally. Positive values indicate the tibial joint line is more varus than the femoral joint line.

1. FNSA

Angle between the femoral neck axis and the femoral anatomical shaft axis.

- Femoral neck axis: line from the femoral head center to the center of the femoral neck (midpoint at smallest cross-section of the femoral neck).
- Femoral anatomical axis: see FAA.
- Measured medially.

1. LDTA

Angle between the tibial anatomical axis and the tangent to the distal tibial articular surface.

- Tibial anatomical axis: see TAA.
- Distal tibial articular surface: line connecting the medial and lateral edges of the distal tibial plafond.
- Measured laterally.

1. TT

Angle between the tibial articular surface and the talar articular surface.

- Tibial articular surface: central line across the distal tibial plafond.
- Talar articular surface: central line connecting the medial and lateral edges of the talar dome.
- Measured medially. Positive values indicate valgus orientation of the talus relative to the tibia.

1. HA

Angle between the talocalcaneal axis and the vertical axis.

- Talocalcaneal axis: line connecting the lowest point of the calcaneus with the center of the talar dome (midpoint between medial and lateral talar edges).
- Vertical reference (Z-axis): perpendicular to the floor.
- Measured posteriorly. Positive values indicate valgus hindfoot alignment; negative indicate varus.

**Axial plane measurements**

1. FAVA

Angle between the femoral neck axis and the posterior condylar line (PCL).

- Femoral neck axis: line from the center of the femoral head to the center of the femoral neck.
- PCL: line connecting the most posterior points of the medial and lateral femoral condyles.
- Measured medially; positive values indicate external rotation (anteversion) of the femoral neck relative to the posterior condylar line.

1. aTEA-PCL

Angle between the anatomical transepicondylar axis and the PCL.

- aTEA: line connecting the most prominent points of the medial and lateral epicondyles.
- PCL: see FAVA.
- Measured medially; positive when the aTEA is externally rotated relative to the PCL.

1. sTEA-PCL

Angle between the surgical transepicondylar axis and the PCL.

- sTEA: line connecting the medial sulcus and the lateral epicondyle.
- PCL: see FAVA.
- Measured medially; positive when the sTEA is externally rotated relative to the PCL.

1. asTEA

Angle between the aTEA and the sTEA.

- aTEA: see aTEA-PCL.
- sTEA: see sTEA-PCL.
- Measured medially; positive when the aTEA is externally rotated relative to the sTEA.

1. TTA-PCL

Angle between the tibial transverse axis and the posterior condylar line.

- Tibial transverse axis (TTA): line connecting the centers of best-fit circles drawn on the medial and lateral tibial plateaus when viewed from axial plane.
- PCL: see FAVA.
- Measured medially; positive when the tibial axis is externally rotated relative to the femoral PCL.

1. FTA-PCL

Angle between the femoral transverse axis and the posterior condylar line.

- Femoral transverse axis (FTA): line connecting the centers of the medial and lateral femoral condyles on the axial slice with the most prominent lateral epicondyle.
- PCL: see FAVA.
- Measured medially; positive values indicate external rotation of the FTA relative to the PCL.

1. FTA-TTA

Angle between the femoral transverse axis and the tibial transverse axis.

- FTA: see FTA-PCL.
- TTA: see TTA-PCL.
- Measured medially; positive when the tibia is externally rotated relative to the femur.

1. FTA-TAPA

Angle between the femoral transverse axis and the trochlear antero-posterior axis (TAPA).

- FTA: see FTA-PCL.
- TAPA: line connecting the cranial and caudal deepest points of the trochlear groove.
- Measured laterally; positive when the TAPA is externally rotated relative to the FTA.

1. TEVA

Angle between the transmalleolar axis and the posterior proximal tibial plateau line.

- Transmalleolar axis: line connecting the most prominent points of the medial and lateral malleoli.
- Posterior proximal tibial plateau line: tangent connecting the most posterior points of the medial and lateral tibial plateaus.
- Measured medially; positive when the transmalleolar axis is externally rotated relative to the proximal tibial plateau.

1. TT-TG

From the PCL, two perpendicular lines are draw, one passing through the center of the tibial tuberosity (TT) and the other through the deepest point of the trochlear groove (TG). The linear distance between these two perpendicular lines represents the TT–TG distance.

- Tibial tuberosity point: most prominent anterior point of the tibial tuberosity in the sagittal/axial view.
- Trochlear groove point: deepest point of the trochlear groove.
- Positive when TT lies lateral to TG.

1. PT

Angle between the patellar transverse axis and the PCL.

- Patellar transverse axis: line that connects the medial and lateral edge of the patella
- PCL: see FAVA
- Measured medially. Positive values indicate increased lateral tilt of the patella relative to the PCL.

1. LTI

Angle between the lateral trochlear surface tangent and the PCL.

- Lateral trochlear surface: straight line that best fits or approximates the slope of the lateral trochlear facet.
- PCL: see FAVA.
- Measured laterally. Positive values indicate internal rotation of the lateral trochlear surface relative to the PCL.

1. SA

Angle formed by connecting the most anterior points of the medial and lateral trochlear ridges with the deepest point of the trochlear groove.

- Measured anteriorly in the axial plane.

**Sagittal plane measurements**

1. MTS

Angle between a line perpendicular to the tibial anatomical axis and a tangent to the medial tibial plateau.

- Tibial anatomical axis: see previous.
- Medial tibial plateau tangent: line connecting the most anterior and most posterior points of the medial tibial plateau on the central sagittal slice.
- Measured posteriorly; positive values indicate a larger posterior inclination (slope) of the medial plateau relative to the shaft axis.

1. LTS

Angle between a line perpendicular to the tibial anatomical axis and a tangent to the lateral tibial plateau.

- Tibial anatomical axis: see previous.
- Lateral tibial plateau tangent: line connecting the most anterior and most posterior points of the lateral tibial plateau on the central sagittal slice.
- Measured posteriorly; positive values indicate a larger posterior inclination (slope) of the lateral plateau relative to the tibial shaft axis.

1. ADTA

Angle between the tibial anatomical axis and the tangent to the distal tibial articular surface in the sagittal plane.

- Tibial anatomical axis: see previous.
- Distal tibial articular surface tangent: line connecting the anterior and posterior edges of the distal tibial plafond.
- Measured anteriorly; positive values indicate a more anteriorly inclined distal tibial joint surface.

1. CPA

Angle between the calcaneal axis and axis parallel to the ground.

- Calcaneal axis: line connecting the lowest point of the calcaneus and the most inferior point on the anterior calcaneal process.
- Measured anteriorly; positive values indicate increased calcaneal inclination.
